# Research Progress in the Regulation of the ABA Signaling Pathway by E3 Ubiquitin Ligases in Plants

**DOI:** 10.3390/ijms25137120

**Published:** 2024-06-28

**Authors:** Hongyun Kou, Xiaopei Zhang, Jinghao Jia, Ming Xin, Jinhui Wang, Lili Mao, Ahmedov Miraziz Baltaevich, Xianliang Song

**Affiliations:** State Key Laboratory of Crop Biology, Agronomy College, Shandong Agricultural University, Tai’an 271018, China

**Keywords:** protein degradation, ubiquitin–proteasome system, E3 ubiquitin ligase, ABA pathway

## Abstract

E3 ubiquitin ligases (UBLs), as enzymes capable of specifically recognizing target proteins in the process of protein ubiquitination, play crucial roles in regulating responses to abiotic stresses such as drought, salt, and temperature. Abscisic acid (ABA), a plant endogenous hormone, is essential to regulating plant growth, development, disease resistance, and defense against abiotic stresses, and acts through a complex ABA signaling pathway. Hormone signaling transduction relies on protein regulation, and E3 ubiquitin ligases play important parts in regulating the ABA pathway. Therefore, this paper reviews the ubiquitin–proteasome-mediated protein degradation pathway, ABA-related signaling pathways, and the regulation of ABA-signaling-pathway-related genes by E3 ubiquitin ligases, aiming to provide references for further exploration of the relevant research on how plant E3 ubiquitin ligases regulate the ABA pathway.

## 1. Introduction

Plants, as sessile organisms, cannot relocate like animals to find suitable habitats. Instead, they have evolved a series of molecular and physiological mechanisms to maximize the reduction of damage caused by both biotic and abiotic stresses [1]. When plants encounter abiotic stresses such as drought, salinity, low temperature, and high temperature, they activate stress-responsive genes to produce various stress proteins to combat the stress. However, the excessive accumulation of abnormal proteins can damage cells, disrupt cellular structures and functions, and affect plant metabolism and growth development. Therefore, a protein degradation system is required to remove abnormal proteins and maintain normal plant growth and development [2].

The ubiquitin/26S proteasome system (UPS) is an important protein degradation pathway in eukaryotic cells involved in finely regulating the growth and development of organisms and their adaptability to the surrounding environment, thereby playing a crucial role in alleviating and reducing damage caused by biotic and abiotic stresses [3]. The UPS comprises several essential components, including ubiquitin molecules (Ub), ubiquitin-activating enzyme E1 (UBA), ubiquitin-conjugating enzyme E2 (UBC), ubiquitin ligase E3 (UBL), and deubiquitinating enzymes (DUBs), as well as proteins involved in substrate recognition and translocation, 26S proteasomes, and ubiquitinated target substrate proteins [4]. Among them, E3 ubiquitin ligases, as key enzymes in the UPS, play a crucial role in recognizing proteins and exert specific recognition during the process of identifying target substrates [5].

Plant responses to external stresses and the maintenance of normal growth development are also regulated by various endogenous hormones. ABA plays an important role in regulating plant growth and development and in defense against biotic and abiotic stresses [6,7]. Since the discovery in 2014 that the ABA receptor PYL8/RCAR3 is regulated by the CUL4-type multi-subunit E3 ligase DET1-, DDB1-ASSOCIATED1 (DDA1), thereby negatively regulating ABA signaling [8], the regulation of the ABA pathway by E3 ubiquitin ligase has gradually become a focus of life science research. Therefore, this paper aims to review the research progress on the regulation of the ABA pathway by E3 ubiquitin ligase in order to provide a reference for subsequent studies.

## 2. The Ubiquitin–Proteasome-Mediated Protein Degradation Pathway

When plants are subjected to biotic and abiotic stressors, proteins within plant cells undergo abnormal expression, leading to impaired cellular function, senescence, and even cell death, which forms an important pathological basis for the occurrence of various diseases [9]. The main protein degradation pathways in eukaryotic cells include the autophagy–lysosome pathway, the mitochondrial calpain pathway, and the ubiquitin–proteasome pathway (UPP). Among these, the UPP is the predominant protein degradation pathway in eukaryotic cells and is responsible for the degradation of 80% of normal or aberrant proteins within cells, serving as a key regulatory factor for cellular homeostasis [10]. Ubiquitin (Ub) is a small molecule widely present in eukaryotic cells, consisting of 76 amino acids with a molecular weight of approximately 8.5 kDa. It is highly conserved in evolution. The full-length ubiquitin molecule contains seven lysine (Lys) residues (K6, K11, K27, K33, K48, and K63), a methionine (Met) residue at the N-terminus (M1), and a glycine (Gly) residue at the C-terminus (G76). Plant ubiquitin differs from animal and yeast ubiquitin by only two and three amino acids, respectively [11].

**Figure 1:**
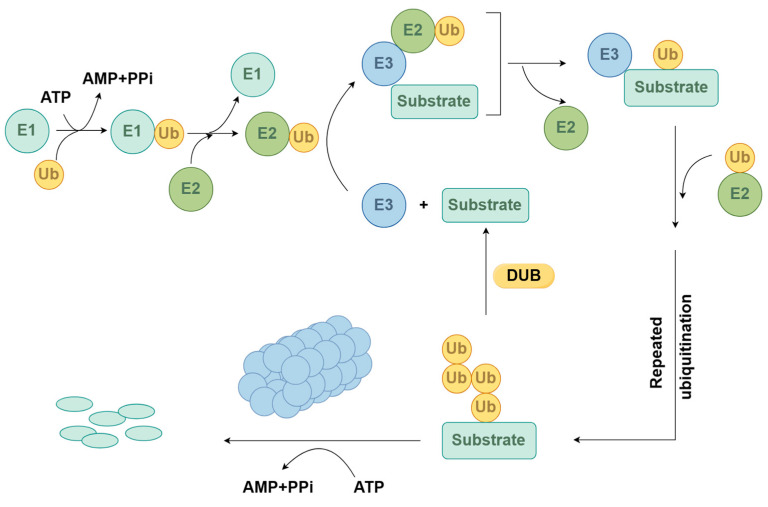
Ubiquitin–proteasome system (by Figdraw). Ubiquitin binds to E1 to form the Ub-E1 complex. The Ub-E1 complex transfers Ub to E2 via a thioesterification reaction, forming the Ub-E2 complex. The Ub-E2 complex transfers Ub to the target protein through two pathways. Finally, most of the ubiquitinated target proteins are degraded by the 26S proteasome, releasing ubiquitin for recycling.

Protein ubiquitination refers to the process of ubiquitin covalently attached to the target protein under the catalysis of a series of enzymes. The “series of enzymes” here refers to three enzymes that synergically participate in the ubiquitination cascade reaction: E1 (ubiquitin-activating enzymes), E2 (ubiquitin-conjugating enzymes), and E3 (ubiquitin ligases) [12]. The transfer of ubiquitin molecules requires the joint participation of E1, E2, and E3 (Figure 1): E1 utilizes the energy provided by ATP to activate ubiquitin molecules to form Ub-E1 complex, which then transfers ubiquitin to E2 through transesterification to form Ub-E2 complex [4]. Ub-E2 complex transfers ubiquitin to target proteins through two pathways. The first pathway involves the direct attachment of the C-terminus of ubiquitin to the Lys residue ε-amino group on the target protein after specific recognition by E3. The second pathway involves transferring ubiquitin to E3 through transesterification, followed by the specific recognition of the target protein by E3, and then attaching the C-terminus of ubiquitin to the Lys residue ε-amino group on the target protein. Finally, most of the ubiquitinated target proteins are degraded by the 26S proteasome, releasing ubiquitin for reuse [13,14]; additionally, some ubiquitinated target proteins are degraded via the autophagy pathway [15]. In summary, under the successive catalysis of E1, E2, and E3 enzymes, ubiquitin is specifically linked to target proteins or to ubiquitin chains already attached to target proteins. Among them, E3 ubiquitin ligase determines the specific recognition of target proteins [16].

The specificity of substrate ubiquitination is primarily determined by the numerous E3 ubiquitin ligases. As enzymes responsible for specifically recognizing target proteins, E3 ligases play a crucial role in protein ubiquitination, making the genes encoding E3 ligases the most abundant among the three enzymes involved in the ubiquitination cascade within the same plant genome. For example, in the genome of *Arabidopsis thaliana*, approximately 1600 genes, accounting for 6% of the total genome, are associated with the UPS pathway. Among them, the genes encoding E1 are the least with only two, while there are at least 37 genes encoding E2 and E2-like enzymes, and over 1500 genes encoding E3 ligases [14,17]. Based on the structure of E3 ubiquitin ligases, they can be divided into two classes: single-subunit and multi-subunit. Single-subunit E3 ligases include the RING (really interesting new gene), U-box (a modified RING motif without the full complement of Zn^2+^-binding ligands) domain, HECT (homologous to E6-associated protein C-terminus), and RBR (ring between ring) types. Multi-subunit E3 ligases mainly include the CRLs family (cullin-RING ligases family) and APC/C type (anaphase-promoting complex/cyclosome) [18,19].

The ubiquitin–proteasome pathway is one of the most important pathways for regulating protein abundance within cells, and E3 ubiquitin ligases, as one of the key components of the UPS, have been demonstrated to take crucial regulatory roles in plant responses to abiotic stress. Plant E3 ubiquitin ligases are involved in various abiotic stress responses, primarily including the regulation of plant immune responses [20,21], the promotion of pollen development [22,23], the enhancement of drought tolerance [24,25,26,27], the improvement of salt tolerance [28,29], the enhancement of cold tolerance [30,31], and the enhancement of heat tolerance [32,33].

## 3. ABA-Related Signaling Pathways

ABA, as one of the nine endogenous hormones in plants, is a plant hormone with a sesquiterpene structure [34]. Natural ABA exists as enantiomers, with the biologically active form being the S-enantiomer (S-ABA) [35,36]. Since its successful discovery by Ohkuma in 1963, the chemical structure, synthesis, and metabolism pathways, as well as the biological functions of ABA, have been reported successively. ABA plays a crucial role in regulating plant growth and development processes [37], such as seed and seedling development, seed dormancy [38], response to pathogens [39], drought [40], high salt, low temperature, and various biotic and abiotic stresses [41].

In 2009, the isolation and functional identification of PYR/PYLs/RCARs-type ABA receptors provided a deeper understanding of the ABA signaling pathway [7,42]. The PYR/PYLs/RCARs family comprises 14 members (PYR1/PYL1-13 or RCAR1-14). It is now widely recognized that PYR/PYLs/RCARs-type receptors play a significant part in the ABA signal transduction pathway [43]. Through the elucidation of protein structures and various biochemical and genetic analyses, it has been revealed that the signaling pathway initiated by PYR/PYLs/RCARs-type receptors involves ABA promoting the inhibition of protein phosphatases PP2Cs (type 2C protein phosphatases) by PYR/PYLs/RCARs, thereby releasing the inhibition of PP2Cs on protein kinase SnRK2s (sucrose non-fermenting 1 (SNF1)-related protein kinase 2s), promoting the phosphorylation modification of SnRK2s substrates, and regulating the expression of ABA-responsive genes [44,45,46]. Various biochemical and omics experiments have identified SnRK2s phosphorylation substrates, including transcription factors located in the nucleus and ion channel proteins on the cell membrane, some of which functionally depend on endocytosis and protein transport processes involving the intracellular membrane system [40,47].

Previous studies using systems biology and other methods have found that ABA signaling networks contain hundreds of interactions between more than 100 proteins, greatly expanding the original ABA signaling pathway. The ABA pathway in plants has now evolved from a simple linear signal to a complex signaling network [48,49]. Among them, the ABA signaling regulatory network (TRAIN) not only includes the traditional PYR/PYL/RCARs→PP2Cs→SnRKs→ABIs/ABFs→target gene signaling pathway, but also direct interactions between PP2Cs and ABIs/ABFs, such as the interaction between PYL8 and the transcription factor MYB49, forming a specific helix–loop–helix structure [48].

## 4. E3 Ubiquitin Ligases Regulate the ABA Signaling Pathway

E3 ubiquitin ligases, as important enzymes in the ubiquitin–proteasome system, participate in various processes of ABA synthesis, signal reception, signal transduction, and downstream factor response in the ABA pathway [50,51,52]. Different E3 ubiquitin ligases finely regulate the ABA signaling pathway by affecting the stability or activity of various ABA-related factors in different subcellular structures. Furthermore, E3 ubiquitin ligases can also influence the ABA pathway by regulating other phytohormones [53].

### 4.1. E3 Ubiquitin Ligases Regulate the ABA Biosynthesis and Degradation Metabolism

ABA is a sesquiterpene compound containing 15 carbon atoms, and plants synthesize ABA via the carotenoid pathway [54,55]. Zeaxanthin epoxidase (ZEP), 9-cis-epoxycarotenoid dioxygenase (NCED), and abscisic-aldehyde oxidase (AAO3) are key enzymes in plants responsible for ABA biosynthesis [56].

The generation of ABA signals is crucial for ABA signal transduction, and E3 ubiquitin ligases can regulate the expression of ABA-synthesis-related genes, thereby modulating ABA levels. The overexpression of *OsRHP1* (encoding a novel RING-H2 finger protein) in rice increases the expression of key enzymes involved in ABA biosynthesis, such as OsZEP, OsNCED, and OsAAO, significantly enhancing rice drought and salt tolerance [57] (Table 1). *XERICO* in *Arabidopsis* encodes a small protein (162 amino acids) with a C-terminal RING-H2 zinc finger domain and induces the upregulation of the ABA biosynthesis gene *AtNCED3* under drought conditions, thereby increasing ABA levels in cells [58]. The U-box type E3 ubiquitin ligase SAUL1/AtPUB44 can interact with ABA aldehyde oxidase AAO3, mediating its ubiquitination via the UPP, leading to a reduction in ABA levels [59].

The dynamic balance of ABA synthesis and degradation metabolism in plants collectively regulates endogenous ABA levels. The ABA 8′-hydroxylation pathway is the main route for endogenous ABA metabolism in higher plants. E3 ubiquitin ligases can regulate the stability of ABA-metabolism-related proteins to modulate ABA levels. In maize, the RING-H2-type E3 ubiquitin ligase *ZmXerico1* interacts with ZmABA8ox1a and ZmABA8ox3a and destabilizes them, thereby reducing ABA degradation and enhancing maize drought tolerance [60].

### 4.2. E3 Ubiquitin Ligases Regulate PYR/PYLs/RCARs Receptor Protein Stability

The reception of the ABA signal is crucial for the transduction of the ABA signaling pathway, with E3 ubiquitin ligases playing a significant role in regulating the reception of the ABA signal.

In *Arabidopsis*, the monomeric RING-type E3 ubiquitin ligase RSL1 (ring finger of seed longevity 1) negatively regulates ABA signaling by promoting the degradation of PYR/PYLs/RCARs-like ABA receptor proteins [61] (Table 2). Conversely, XBAT35 degrades the inner membrane sorting complex component VPS23A, releasing VPS23A’s inhibitory effect on the PYL4 protein, thereby aiding in the activation of the ABA signaling pathway to cope with environmental stresses [52]. U-box type ubiquitin ligases PUB22 and PUB23 interact with the ABA receptor PYL9, leading to the degradation of RCAR1, negatively regulating plant drought tolerance [24]. PUB11 negatively regulates drought tolerance by degrading the receptor-like protein kinases LRR1 and KIN7 [62]. RBR-type E3 ligases RFA1 and RFA4, after interaction with the E2 conjugating enzyme UBC26, bind to ABA receptors such as PYR1 (RCAR11), PYL4 (RCAR10), PYL5 (RCAR8), and PYL8 (RCAR3), thereby negatively regulating ABA signaling by controlling ABA receptor stability [63]. The multi-subunit CULLIN-RING-type E3 ubiquitin ligase substrate receptor protein DDA1 mediates the ubiquitination of PYL8/RCAR3 receptors, negatively regulating ABA signaling [8]. RIFP1 is a subunit of the SCF (SKP1/Cullin/F-box) complex E3 ligase, which ubiquitinates RCAR3, thus regulating RCAR3 levels after drought stress [64]. The CUL4-DDB1-DWD-type E3 ubiquitin ligase AtRAE1 similarly ubiquitinates RCAR1 and negatively regulates the ABA signaling pathway [65].

### 4.3. E3 Ubiquitin Ligases Regulate PP2Cs Phosphatase Activity

In addition to regulating the reception of ABA signals, E3 ubiquitin ligases also participate in ABA signal transduction. PP2C-type phosphatases serve as co-receptors for ABA signal reception, responding to ABA signals alongside PYR/PYLs/RCARs-type ABA receptors [66]. The PP2C-type phosphatase ABI1 protein can be degraded by the 26S proteasome. Two members of the U-box family, PUB12 and PUB13, participate in the process of 26S proteasome-mediated degradation of the ABI1 protein, with PUB12 and PUB13 only ubiquitinating ABI1 in the presence of both ABA and PYR1 [67] (Table 3).

In *Arabidopsis*, the RING-type E3 ubiquitin ligase RGLG1 can respond to increased ABA and its target levels, altering its subcellular localization and promoting nuclear interaction with PP2CA, therefore enhancing PP2CA degradation [68]. RGLG5, by mediating the ubiquitination degradation of PP2CA, promotes the activation mechanism of the ABA signaling pathway, enhancing *Arabidopsis* drought tolerance [69]. PIR1 (PP2CA-interacting RING finger protein 1) and PIR2 positively regulate ABA signal transduction by targeting the degradation of PP2CA [70]. AIRP3 can form an E2–E3 pair with the E2-conjugating enzyme UBC27, and both AIRP3 and UBC27 interact with ABI1, affecting the ubiquitination and degradation of ABI1 [71]. COP1 interacts with ABI/HAB and AHG3, leading to their ubiquitination, and is a key participant in ABA signal transduction, providing a new pathway for regulating stomatal closure in *Arabidopsis* [72]. In pepper (*Capsicum annuum*), the E3 ligase CaAIRF1 interacts with CaADIP1 and ubiquitinates it, positively regulating ABA signaling [73]. OsRF1 (RING finger E3 ligase 1) in rice is a novel small RING-H2 type E3 ligase, positively regulating ABA signal transduction by targeting the degradation of OsPP2C09, increasing ABA biosynthesis, and, thus, responding to drought and salt stress [74]. Different E3 ubiquitin ligases affect ABA signaling by influencing PP2Cs activity.

### 4.4. E3 Ubiquitin Ligases Regulate SnRK2s Kinase Activity

The transition of SnRK2s from an inactive dephosphorylated state to an activated state within plant cells is a crucial step in the response to ABA and environmental stress. The regulation of SnRK2s kinase activity by E3 ubiquitin ligases significantly impacts ABA signal transduction.

In *Arabidopsis*, AtPP2-B11 is a part of the SCF E3 ubiquitin ligase complex and can specifically promote the degradation of SnRK2.3 to negatively regulate the plant response to ABA [75] (Table 4). HOS15 is a substrate receptor of the CUL4-DDB1-type E3 ubiquitin ligase, which interacts specifically with OST1, SnRK2.3, and ABI1/2. HOS15 negatively regulates ABA signal transduction and drought stress resistance by interfering with the stability of OST1 [76]. In cucumber, a RING-type E3 ligase, CsCHYR1, exhibits auto-ubiquitination activity. CsCHYR1 interacts with CsSnRK2.6 and CsATAF1, forming a regulatory model, CsSnRK2.6-CsCHYR1-CsATAF, which regulates plant response to drought stress through an ABA-dependent pathway [51].

### 4.5. E3 Ubiquitin Ligases Regulate ABIs/ABFs and Other Downstream Response Transcription Factors

ABA signals are transmitted sequentially and eventually reach the nucleus, activating or inhibiting the activity of various transcription factors to initiate the expression of downstream response genes [77]. Different types of E3 ubiquitin ligases can regulate ABA response gene expression either positively or negatively, thereby influencing ABA pathway transduction.

#### 4.5.1. RING Type

In *Arabidopsis*, the RING-type E3 ligase AtAIRP3/LOG2 (Lonely Guy 2) interacts with RD21 and acts as a positive regulator of ABA signaling, promoting UPS-dependent degradation of ABI5 and other bZIP TFs downstream [78,79] (Table 5). ABI3 can be degraded by the 26S proteasome, and AIP2 (ABI3-Interacting Protein2) interacts with ABI3 and FUS3 (FUSCA3), mediating the ubiquitination of ABI3 both in vivo and in vitro, thus negatively regulating ABA signaling [80,81,82]. The accumulation of AtMBP-1 is limited by ubiquitin-dependent instability, which is regulated by interaction with AtSAP5. The E3 ubiquitin ligase AtSAP5 promotes UPS degradation of AtMBP-1 upstream of ABI5, ABI3, and ABI4 [83]. *Arabidopsis* ATLs comprise a special group of RING-type E3 ubiquitin ligases involved in abiotic stress responses. The transcription of ATL78 is regulated by ABA as well as drought, salt, and cold stress. ATL78 modulates ABA-dependent stomatal closure and the accumulation of reactive oxygen species (ROS) during drought stress, positively regulating the ABA signaling pathway [84,85]. SDIR1 is an endoplasmic reticulum membrane-localized ABA-positive regulator that interacts with SDIRIP1 (SDIR1-interacting protein 1) and ubiquitinates it, and then regulates the stability of SDIRIP1 via the UPP [86]. Moreover, SDIRIP1 can be ubiquitinated by AIRP2 as a target, further positively responding to ABA signaling [87]. In wild-type *Arabidopsis*, the E3 ubiquitin ligase AtAIRP5/GARU plays a role as a positive regulator of ABA-mediated drought response by promoting the degradation of AtSCPL1 (serine carboxypeptidase-like1) [88]. The protein kinase CIPK26 serves as a ubiquitination substrate of the E3 ubiquitin ligase KEG (keep on going), which negatively regulates the ABA signal by mediating the degradation of CIPK26 via the proteasome pathway [89]. Moreover, KEG may also mediate the ubiquitination degradation of bZIP TFs ABI5, ABF1, and ABF3 [90]. MYB96 can directly regulate the expression of ABI4, while MIEL1 negatively regulates ABA sensitivity by promoting the turnover of MYB96 [91,92]. MIEL1 also directly mediates the proteasomal degradation of ABI5 and inhibits its activity by releasing its target protein MYB30, thus precisely controlling ABA signal transduction during seed germination and seedling establishment [93]. Moreover, in *Arabidopsis*, there exists a E3 ubiquitin ligase SINA2 (SEVEN IN ABSENTIA 2), lacking the classical RING domain but containing a B-box domain, which interacts with a nuclear protein CDKG1 (cyclin-dependent kinase G1) and positively regulates plant response to ABA and osmotic stress [94].

In addition to *Arabidopsis*, E3 ubiquitin ligase has also been reported to be involved in regulating the ABA pathway in other plants. In rice, the RING-type E3 ligase OsCTR1 mediates drought tolerance by targeting the degradation of OsCP12 and OsRP1 and regulating their subcellular transport in an ABA-dependent manner [95]. In wheat, the RING-HC zinc finger protein TaDIS1 mediates the drought stress response by interacting with TaSTP, and the overexpression of TaDIS1 leads to increased sensitivity to ABA [96]. TaSDIR1-4A encodes a C3H2C3-type RING E3 ligase that mediates the C-terminal ubiquitination and proteolysis of TaWRKY29, transporting it from the plasma membrane to the nucleus. Activated TaWRKY29 binds to the TaABI5 promoter, stimulating its expression, positively regulating the ABA signaling pathway and drought response [97]. In chili pepper, CaASRF1 positively regulates ABA signal transduction and drought stress response by modulating the stability of CaAIBZ1 and CaATBZ1 [98,99]. Furthermore, the stability of CaATBZ1 mediated by CaATIR1 plays a crucial role in drought stress signal transduction. By degrading CaAIBZ1 and CaATBZ1, it positively regulates ABA signal transduction and enhances drought tolerance [90]. In maize, the RING-H2 type E3 ubiquitin ligase ZmWIPF2 interacts with ZmCCT, negatively regulating ABA signaling [100].

#### 4.5.2. U-Box Type

In *Arabidopsis*, U-box E3 ligase AtPUB9 is a nucleus-localized negative regulator of ABA and plays a role upstream of ABI3 [101]. PUB8 interacts with ABI3 and ABI5 and negatively regulates ABA responses during early growth of *Arabidopsis* seedling [102]. In rice, MODD (Mediator of OsbZIP46 deactivation and degradation) interacts with the OsPUB70 to promote the degradation of OsbZIP46 and inhibit the expression of OsbZIP46 target genes, negatively regulating ABA signal transduction and drought resistance [103]. On the other hand, OsHUB2, an E3 ligase for H2Bub1, interacts with OsbZIP46 as a positive regulator of ABA signal transduction and drought response [104]. Apple MdABI5, through the MdABI5-MdbHLH93-MdSAG18 regulatory module, accelerates leaf senescence, while U-box-type E3 ubiquitin ligase MdPUB23 interacts with MdABI5 to delay leaf senescence triggered by ABA [50]. In addition, CaPUB24 in chili negatively regulates drought stress response in an ABA-dependent manner, but the specific genes in the ABA signaling pathway it regulates are not known [105].

#### 4.5.3. HECT Type

The RING-type E3 ligases have been extensively studied in different stages of ABA signaling, while HECT-type E3 ligases have been least studied in various stages of ABA signaling. In *Arabidopsis*, the HECT-type UPL1 and UPL4, as well as the RING-type KEG, can ubiquitinate MAPKKK17/18 and promote their degradation, negatively regulating the ABA signal [106].

#### 4.5.4. CRLs Type

*Arabidopsis* DWA1/DWA2 (DWD is allergic to ABA1/2) encodes a substrate receptor for CUL4 type E3 ligase, interacts with ABI5 in vivo, and targets its degradation, thereby negatively regulating ABA signaling [107]. ABD1 (ABA-hypersensitive DCAF1), a component of the DCAF-CUL4-type E3 ubiquitin ligase complex, directly binds to ABI5 and influences its stability in the nucleus, negatively modulating *Arabidopsis* ABA signaling [108]. MATH-BTB proteins are substrate-binding receptors for Cullin3-based E3 ubiquitin ligases. MATH-BTB proteins directly interact with the transcription factor ATHB6, targeting it for proteasomal degradation and positively regulating ABA signaling [109].

**Figure 2:**
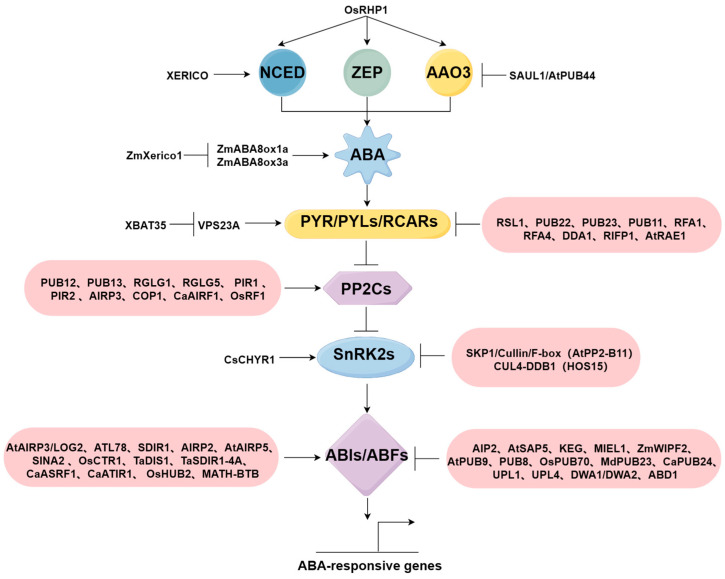
E3 ubiquitin ligase regulation of the ABA pathway (by Figdraw). NCED, ZEP, and AAO3 are key enzymes in ABA biosynthesis. The pink boxes represent the regulatory effects of different E3 ubiquitin ligases on components such as PYR/PYLs/RCARs, PP2Cs, SnRK2s, and ABIs/ABFs in the ABA pathway. Arrows and vertical bars with horizontal lines represent positive regulation and negative regulation, respectively.

There are numerous E3 ubiquitin ligases in the plant genome, and the different types of E3 mentioned above regulate ABA signaling by affecting the expression of related genes at different stages of the ABA signaling pathway (Figure 2), which helps plants to better adapt to the adverse environment and maintain normal growth and development.

## 5. Conclusions and Outlook

Throughout their lifecycle, plants constantly face abiotic stresses. To cope with a range of stress responses, plants have evolved corresponding defense mechanisms to maintain normal growth and development. E3 ubiquitin ligases, as vital components of the ubiquitin–proteasome pathway, comprise a large and functionally complex family. They are currently a focal point in the study of plant growth, development, and stress response mechanisms. ABA is a crucial phytohormone in plants for combating environmental stresses. While the core signaling pathway model of ABA has been established, the ABA signaling transduction network is quite complex, and the response mechanisms of its signal pathways still need refinement. To further unravel the defense mechanisms of plants against abiotic stresses, research on E3 ubiquitin ligases in the ABA signaling pathway is indispensable. While a significant number of E3 ubiquitin ligases have been found to play important roles in the ABA pathway, only a few have been fully elucidated. Many functions and mechanisms of E3 ligases remain unclear, especially for non-RING types, which have been less studied. Future research will continue to explore various aspects of this field. With the in-depth study of the ABA signaling pathway, it is believed that more E3 ubiquitin ligases will be discovered and analyzed in depth. We still need to employ a plethora of methods such as molecular biology, genetics, protein–protein interactions, and omics to uncover the specific targets and regulatory mechanisms of different types of E3 ubiquitin ligases in the ABA signaling pathway. This analysis aims to elucidate their roles in plant growth, development, and response to environmental stresses, laying the groundwork for the development of crops with higher tolerance to environmental stress.

Additionally, E3 ubiquitin ligases can also regulate other endogenous hormone pathways, contributing to plant defense against abiotic stresses by modulating the interactions between other endogenous hormones and ABA. Current research has reported on E3 ubiquitin ligases regulating the interaction between ABA and JA (jasmonic acid) [53] and GA (gibberellic acid) [110] in plant biological processes, but the functional and mechanistic analyses are still incomplete. In future studies, we should strengthen research in this area using new tools and methods. Simultaneously, the development of new drugs targeting plant E3 ubiquitin ligases can be explored to regulate plant growth, development, and stress resistance, providing new strategies to enhance plant stress resilience and improve crop yield and quality. In conclusion, future research will further reveal the important role of plant E3 ubiquitin ligases in the ABA pathway, providing new insights into understanding plant growth, development, and adaptation to the environment, and offering innovative solutions for agricultural production.

## Figures and Tables

**Table 1:** E3 ubiquitin ligases regulate ABA biosynthesis and degradation metabolism.

E3 Type	Protein or Complex	Targets	Organism	Regulation of ABA	References
RING-H2	OsRHP1	OsZEP, OsNCED, OsAAO	Rice	Positive	[57]
	XERICO	NCED3	*Arabidopsis*	Positive	[58]
	ZmXerico1	ZmABA8ox1a, ZmABA8ox3a	Maize	Positive	[60]
U-box	SAUL1/AtPUB44	AAO3	*Arabidopsis*	Negative	[59]

**Table 2:** E3 ubiquitin ligases regulate PYR/PYLs/RCARs receptor protein stability.

E3 Type	Protein or Complex	Targets	Organism	Regulation of ABA	References
RING	RSL1	PYR/PYLs/RCARs	*Arabidopsis*	Negative	[61]
	XBAT35	VPS23A	*Arabidopsis*	Positive	[52]
U-box	PUB22/PUB23	PYL9	*Arabidopsis*	Negative	[24]
	PUB11	LRR1/KIN7	*Arabidopsis*	Negative	[62]
RBR	RFA1/RFA4	PYR1/4/5/8	*Arabidopsis*	Negative	[63]
CRLs	CRL4_DDA1	PYL8/RCAR3	*Arabidopsis*	Negative	[8]
	SCF_RIFP1	RCAR3	*Arabidopsis*	Negative	[64]
	AtRAE1	RCAR1	*Arabidopsis*	Negative	[65]

**Table 3:** E3 ubiquitin ligases regulate PP2Cs phosphatase activity.

E3 Type	Protein or Complex	Targets	Organism	Regulation of ABA	References
U-box	PUB12/PUB13	ABI1	*Arabidopsis*	Positive	[67]
RING	RGLG1	PP2CA	*Arabidopsis*	Positive	[68]
	RGLG5	PP2CA	*Arabidopsis*	Positive	[69]
	PIR1/PIR2	PP2CA	*Arabidopsis*	Positive	[70]
	AIRP3	ABI1	*Arabidopsis*	Positive	[71]
	COP1	ABI/HAB, AHG3	*Arabidopsis*	Positive	[72]
	CaAIRF1	CaADIP1	Capsicum annuum	Positive	[73]
	OsRF1	OsPP2C09	Rice	Positive	[74]

**Table 4:** E3 ubiquitin ligases regulate SnRK2s kinase activity.

E3 Type	Protein or Complex	Targets	Organism	Regulation of ABA	References
CRLs	SCF_AtPP2-B11	SnRK2.3	*Arabidopsis*	Negative	[75]
	CUL4-DDB1-HOS15	OST1	*Arabidopsis*	Negative	[76]
RING	CsCHYR1	CsSnRK2.6, CsATAF1	Cucumber	Positive	[51]

**Table 5:** E3 ubiquitin ligases regulate ABIs/ABFs and other downstream response transcription factors.

E3 Type	Protein or Complex	Targets	Organism	Regulation of ABA	References
RING	AtAIRP3/LOG2	RD21	*Arabidopsis*	Positive	[78]
	AIP2	ABI3, FUS3	*Arabidopsis*	Negative	[80]
	AtSAP5	AtMBP-1	*Arabidopsis*	Negative	[83]
	ATL78	Catalases	*Arabidopsis*	Positive	[85]
	SDIR1	SDIRIP1	*Arabidopsis*	Positive	[86]
	AIRP2	SDIRIP1	*Arabidopsis*	Positive	[87]
	AtAIRP5/GARU	AtSCPL1	*Arabidopsis*	Positive	[88]
	KEG	CIPK26, bZIP TFs	*Arabidopsis*	Negative	[89]
	MIEL1	MYB96, ABI5	*Arabidopsis*	Negative	[91,93]
	SINA2	CDKG1	*Arabidopsis*	Positive	[94]
	OsCTR1	OsCP12, OsRP1	Rice	Positive	[95]
	TaDIS1	TaSTP	Triticum aestivum	Positive	[96]
	TaSDIR1-4A	TaWRKY29	Triticum aestivum	Positive	[97]
	CaASRF1	CaAIBZ1, CaATBZ1	Capsicum annuum	Positive	[98,99]
	CaATIR1	CaAIBZ1, CaATBZ1	Capsicum annuum	Positive	[90]
	ZmWIPF2	ZmCCT	Maize	Negative	[100]
U-box	AtPUB9	ABI3	*Arabidopsis*	Negative	[101]
	PUB8	ABI3, ABI5	*Arabidopsis*	Negative	[102]
	OsPUB70	OsbZIP46	Rice	Negative	[103]
	OsHUB2	OsbZIP46	Rice	Positive	[104]
	MdPUB23	MdABI5	Apple	Negative	[50]
	CaPUB24	ND	Capsicum annuum	Negative	[105]
HECT	UPL1/UPL4	MAPKKK17/18	*Arabidopsis*	Negative	[106]
CRLs	DWA1/DWA2	ABI5	*Arabidopsis*	Negative	[107]
	ABD1	ABI5	*Arabidopsis*	Negative	[108]
	MATH-BTB	ATHB6	*Arabidopsis*	Positive	[109]

ND: not detected.

## Data Availability

Not applicable.
